# Increased Risk of Thrombocytopenia and Death in Patients with Bacteremia Caused by High Alpha Toxin-Producing Methicillin-Resistant *Staphylococcus aureus*

**DOI:** 10.3390/toxins13100726

**Published:** 2021-10-14

**Authors:** Fatimah Alhurayri, Edith Porter, Rachid Douglas-Louis, Emi Minejima, Juliane Bubeck Wardenburg, Annie Wong-Beringer

**Affiliations:** 1 College of Clinical Pharmacy, Imam Abdulrahman Bin Faisal University, Dammam 31441, Saudi Arabia; Faalhurayri@iau.edu.sa; 2 School of Pharmacy, University of Southern California, Los Angeles, CA 90033, USA; rachiddo@usc.edu (R.D.-L.); minejima@usc.edu (E.M.); 3 College of Natural & Social Sciences, California State University Los Angeles, Los Angeles, CA 90032, USA; eporter@exchange.calstatela.edu; 4 School of Medicine, Washington University, St. Louis, MO 63130, USA; jbubeck@wustl.edu; 5 Department of Pharmacy, Huntington Memorial Hospital, Pasadena, CA 91105, USA

**Keywords:** alpha toxin, virulence factors, *Staphylococcus aureus* bacteremia, mortality, platelets, thrombocytopenia

## Abstract

Alpha toxin (Hla) is a major virulence factor of *Staphylococcus aureus* that targets platelets but clinical data on Hla pathogenesis in bacteremia (SAB) is limited. We examined the link between in vitro Hla activity and outcome. Study isolates obtained from 100 patients with SAB (50 survivors; 50 non-survivors) were assessed for in vitro Hla production by Western immunoblotting in a subset of isolates and Hla activity by hemolysis assay in all isolates. Relevant demographics, laboratory and clinical data were extracted from patients’ medical records to correlate Hla activity of the infecting isolates with outcome. Hla production strongly correlated with hemolytic activity (*r_s_* = 0.93) in vitro. A trend towards higher hemolytic activity was observed for MRSA compared to MSSA and with high-risk source infection. Significantly higher hemolytic activity was noted for MRSA strains isolated from patients who developed thrombocytopenia (median 52.48 vs. 16.55 HU/mL in normal platelet count, *p* = 0.012) and from non survivors (median 30.96 vs. 14.87 HU/mL in survivors, *p* = 0.014) but hemolytic activity of MSSA strains did not differ between patient groups. In vitro Hla activity of *MRSA* strains obtained from patients with bacteremia is significantly associated with increased risk for thrombocytopenia and death which supports future studies to evaluate feasibility of bedside phenotyping and therapeutic targeting.

## 1. Introduction

*Staphylococcus aureus* is a leading cause of bloodstream infection, affecting an estimated 50 in 100,000 people annually with an overall mortality rate of up to 57% in adults [1]. Despite receipt of antibiotic therapy, one in three patients develop persistent bacteremia, which is associated with complications, prolonged hospitalization, and increased risk of death [2]. Numerous factors may contribute to the varied outcomes observed in *S. aureus* bacteremia (SAB) including heterogeneity in host immunity and variable expression of virulence factors across clinical *S. aureus* strains [1].

Alpha toxin (Hla), a water-soluble, 34 kDa monomer, is a well-characterized cytolysin that is known to play a key role in the pathogenesis of *S. aureus* infections. Upon binding of Hla to its host cell receptor, ADAM10 (A Disintegrin And Metalloproteinase domain-containing protein-10) which is widely expressed on endothelial cells, epithelial cells, and immune cells, oligomerization of the toxin occurs leading to heptameric pores in the membranes and subsequent alterations in cellular signaling and cellular lysis [3]. Platelets are the most abundant, non-nucleated immune cells in circulation and have been shown to kill *S. aureus* directly and indirectly through functional enhancement of other immune cells such as macrophages [4]. Wuescher et al. found reduced survival rate with increased cytokine storm and higher bacterial load in kidneys of platelet-depleted mice infected with USA300 causing bacteremia compared to wild-type (WT) mice without platelet depletion [5]. Importantly, recent studies have demonstrated that Hla targets platelets and causes platelet activation, aberrant aggregation, and injury [6,7,8]. Surewaard et al. found more thrombocytopenia and significantly higher platelet aggregation in the livers of mice that were infected with wild-type *S. aureus* compared to an isogenic *hla*-deletion mutant [8]. Additionally, Hla was shown to induce platelet desialylation resulting in enhanced and premature platelet clearance through the hepatic Ashwell-Morell receptor (AMR) [7]. High Hla production was shown to increase severity and reduce survival of infection in numerous experimental models of pneumonia [9,10], sepsis [6], peritonitis [11], and brain abscesses [12]. Administration of a novel anti-alpha toxin monoclonal antibody developed to specifically target and neutralize Hla was shown to provide survival benefit in multiple animal models of infection, including pneumonia [13,14], skin and soft-tissue infections [15,16] and bacteremia [17].

Despite the extensive research that highlights Hla as a major toxin in animal models and the considerable efforts towards developing antibodies that target and neutralize Hla in *S. aureus* infections, few studies assessed the relationship between in vitro Hla production by clinical isolates as a possible predictive marker for the development of thrombocytopenia and patient outcomes in SAB. Given the previously reported variation in Hla expression across *S. aureus* strains [18,19,20] and its involvement in the pathogenesis of *S. aureus* infections, we hypothesized that *S. aureus* bloodstream isolates produce varying Hla levels and that high Hla-producing strains are associated with increased risk of thrombocytopenia and mortality in patients with bacteremia. Our study objectives were to: (1) measure the in vitro level of Hla production and hemolytic activity of *S. aureus* bloodstream isolates and (2) correlate Hla activity with platelet count and outcome of SAB. 

## 2. Results

### 2.1. Patient Characteristics and Platelet Trends

Table 1 compares the characteristics between those with and without thrombocytopenia and between survivors and non-survivors in terms of sex, age, IV drug use, and comorbid conditions that may predispose to the development of thrombocytopenia, cause and source risk of infection, and platelet count at onset. Thrombocytopenia occurred in 36% (34/95) of our study cohort overall, with 8-times greater proportion among non-survivors than survivors (57%, 27/47 vs. 14%, 7/48; *p* < 0.0001, OR 7.91, CI: 2.89 to 19.44). Among those who developed thrombocytopenia at onset of SAB, a greater proportion had sources of infection associated with high risk for death such as endovascular and lower respiratory tract infections (50% vs. 24%, *p* = 0.022) and had MRSA as a causative pathogen (53% vs. 31%, *p* = 0.048) when compared to those who did not, though the groups did not differ in age or comorbid conditions except for liver cirrhosis (24% vs. 8%, *p* = 0.059). On the other hand, non-survivors were older (median 62 vs. 52 years, *p* = 0.002) with a trend towards greater proportion with active malignancy (20% vs. 6%, *p* = 0.071). Similarly, non-survivors were also more likely to have high risk source infections (44% vs. 22%, *p* = 0.032). Of the 95 patients with available platelet counts at onset of SAB, the median platelet counts were 96.5, 241, 221, 128 × 10^9^/L in the following groups, respectively: thrombocytopenic, non-thrombocytopenic, survivors and non-survivors. 

**Figure 1:**
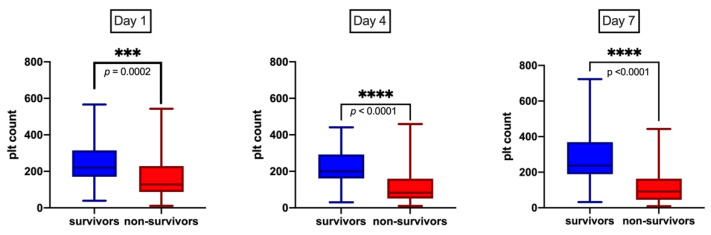
Association between platelets dynamics during SAB. Strong positive association between serial measurements of platelets (plt) counts during first 7 days of bacteremia and survival. *p* values as determined by Mann–Whitney tests. *** and ****: level of significance.

An analysis of platelet dynamics during the first 7 days of bacteremia showed a decline of platelet counts through day 4 with negligible recovery among non-survivors relative to survivors (median platelet count, 1 × 10^9^/L): on day 1 [128 (IQR 87, 229) vs. 221 (IQR 170, 315); *p* = 0.0002], on day 4 [83 (IQR 51,159 vs. 200 (IQR 161, 292); *p* < 0.0001)], and on day 7 [91 (IQR 44.7, 163) vs. 237 (IQR 189, 369); *p* <0.0001)] (Figure 1).

### 2.2. High Correlation of Hla Protein Level and Hemolytic Activity 

**Figure 2:**
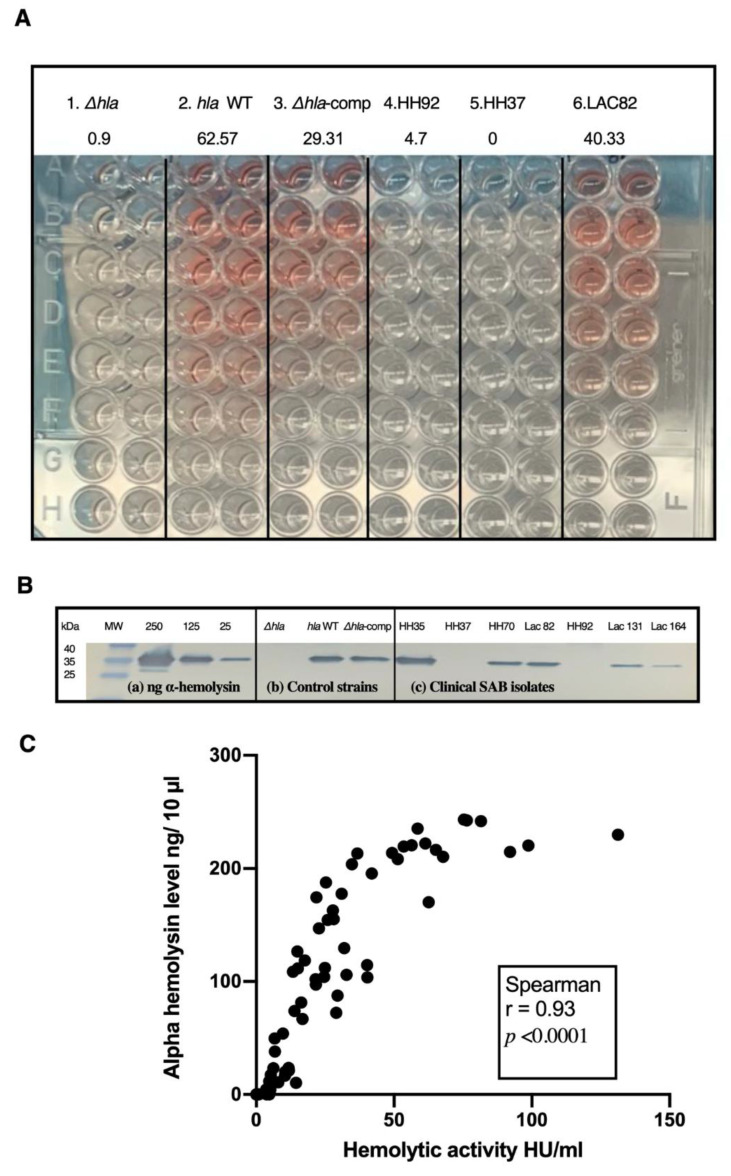
Correlation of Western immunoblot and hemolysis assays. (**A**) Representative image of hemolysis assay with supernatants that were tested in duplicates, collected from control strains (1. isogenic Δ*hla* mutant lacking Hla production, 2. LAC (USA300) *hla* wild type and 3. Δ*hla*-complemented mutant strains with restored Hla production) and clinical isolates (4. HH92, 5. HH37, and 6. LAC82) in a 96 well plate. Hla expression (in hemolytic units per ml, HU/mL). (**B**) Western immunoblot for (**a**) known amounts of purified Hla, (**b**) control strains and (**c**) representative SAB clinical isolates. (**C**) Correlation between Hla protein concentration and hemolytic activity, *p* values as determined by Spearman correlation test.

We evaluated Hla expression by first determining the correlation between protein levels and hemolytic activity measured by Western immunoblot and hemolysis assays, respectively, in a subset of 61 clinical isolates, representing survivors and non-survivors, MRSA and MSSA isolates, plus the three control strains (Figure 2). A very strong correlation was found between Hla protein level and hemolytic activity (*r_s_* = 0.93, *p* < 0.0001). Based on these findings and considering the labor intensity of the Western immunoblot, we performed hemolysis assays only on the remaining clinical isolates and all subsequent analysis was based on results obtained from the hemolysis assay. 

### 2.3. Association of High Hemolytic Activity with Thrombocytopenia and Mortality 

**Figure 3:**
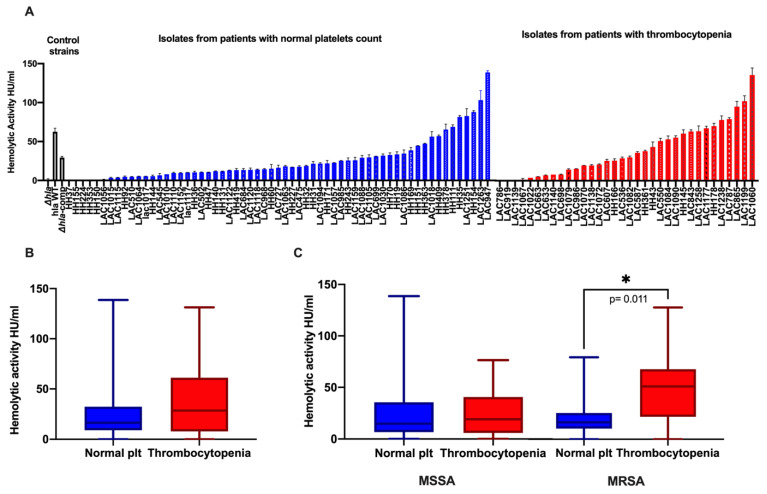
Hemolytic activity of *S. aureus* isolates by platelet status of patients. Hemolytic activity (HU/mL) for each isolate is represented by the average value measured from two independent hemolysis assays performed in duplicates. For the different group comparisons, the box plots represent the median value (HU/mL) with interquartile range (IQR) and the whiskers represent the max and min values. *p* values as determined by Mann–Whitney test. (**A**) Distribution of hemolytic activity across SAB isolates grouped by platelet status. Control strains: LAC (USA300) *hla* wild type, isogenic Δ*hla* mutant lacking Hla production, and Δ*hla*-complemented mutant strains with restored Hla production. (**B**) Comparison of *S. aureus* hemolytic activity from patients with thrombocytopenia vs. those with normal platelet count (plt) (including both MSSA and MRSA): median 28.1 HU/mL (IQR 7.62, 61.1) vs. 16.34 HU/mL (IQR 8.8, 32.3); *p =* 0.08]. (**C**) Comparison of *S. aureus* hemolytic activity from patients with normal platelet count and thrombocytopenia grouped by MSSA or MRSA. MRSA isolates from patients with thrombocytopenia vs. normal platelet count [median 52.48 HU/mL (IQR 22.19, 69.71) vs. 16.55 HU/mL (IQR 9.8, 26.82), *p* = 0.011]. *: Statistical significance.

**Figure 4:**
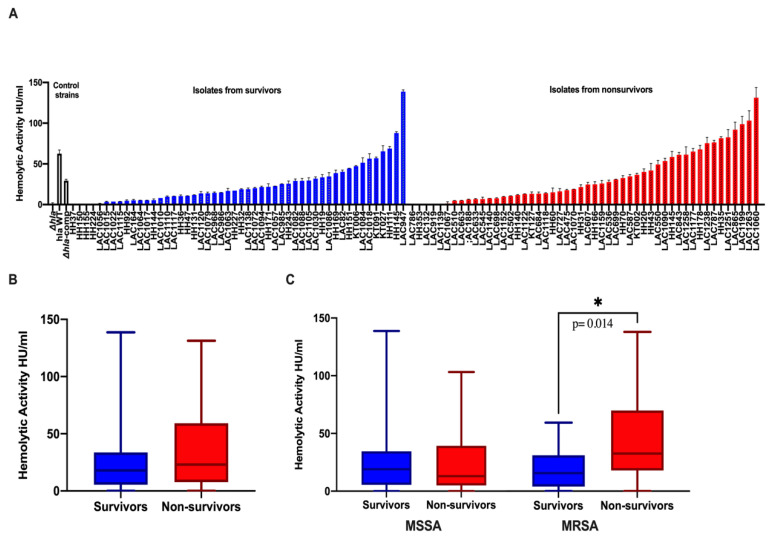
Hemolytic activity of *S. aureus* isolates by survival status of patients. Hemolytic activity (HU/mL) for each isolate is represented by the average value measured from two independent hemolysis assays performed in duplicates. For the different group comparisons, the box plots represent the median value (HU/mL) with interquartile range (IQR) and the whiskers represent the max and min values. *p* values as determined by Mann–Whitney test. (**A**) Distribution of alpha hemolysin hemolytic activity across SAB isolates grouped by survival status. Control strains: LAC (USA300) *hla* wild type, isogenic Δ*hla* mutant lacking Hla production, and Δ*hla*-complemented mutant strains with restored Hla production. (**B**) Comparison of *S. aureus* hemolytic activity from non-survivors and survivors (including both MSSA and MRSA): [median 23.1 HU/mL (IQR 7.62, 59.2) vs. 17.97 HU/mL (IQR 5.42, 33.73); *p =* 0.252]. (**C**) Comparison of *S. aureus* bloodstream isolates hemolytic activity from survivors and non-survivors grouped by MSSA or MRSA: MRSA-infected patients who died vs. those who survived [median 30.96 HU/mL (IQR 17, 66.50) vs. 14.87 HU/mL (IQR 3.76, 29.53), *p =* 0.014]; MSSA-infected patients who died vs. those who survived [median 13 HU/mL (IQR 4.91, 39.32) vs. 18.99 HU/mL (IQR 5.45, 34.50), *p* = 0.353]. *: Statistical significance.

Bloodstream *S. aureus* isolates exhibited a wide variation of Hla expression in terms of hemolytic activity (range: 0 to 138.7 HU/mL). Distribution of hemolytic activity for *S. aureus* isolates are shown grouped by platelet status (Figure 3A) and survival status of patients (Figure 4A). Overall, higher hemolytic activity was observed for MRSA vs. MSSA isolates (25.12 vs. 14.76 HU/mL, *p* = 0.09) (data not shown). Similarly, a trend towards higher hemolytic activity was observed for isolates from patients with thrombocytopenia vs. those from patients with normal platelet count [median 28.1 HU/mL (IQR 7.62, 61.1) vs. 16.34 HU/mL (IQR 8.8, 32.3); *p =* 0.08] (Figure 3B), and from non-survivors vs. survivors [median 23.1 HU/mL (IQR 7.62, 59.2) vs. 17.97 HU/mL (IQR 5.42, 33.73); *p =* 0.252] (Figure 4B). When isolates were grouped based on methicillin resistance, a striking difference in hemolytic activity was observed for MRSA isolates from patients with thrombocytopenia vs. normal platelet count [median 52.48 HU/mL (IQR 22.19, 69.71) vs. 16.55 HU/mL (IQR 9.8, 26.82), *p* = 0.011] (Figure 3C) and from patients who died vs. those who survived [median 30.96 HU/mL (IQR 17, 66.50) vs. 14.87 HU/mL (IQR 3.76, 29.53), *p.=* 0.014) (Figure 4C). 

On the contrary, MSSA isolates exhibited similar Hla activity regardless of platelet count status of the infected patients [median 14.76 HU/mL (IQR 6.4, 35.5) vs. 18.9 HU/mL (IQR 5.7, 40.6); *p* = 0.91] (Figure 3C) or survival status [median 18.99 HU/mL (IQR 5.45, 34.50) vs. 13 HU/mL (IQR 4.91, 39.32), *p* = 0.353] (Figure 4C). Notably, the mean platelet count among MRSA-infected group was significantly lower compared to MSSA group [155.8 +/− 87.98 SD vs. 237 +/− 125.6 SD, *p* = 0.0008] (data not shown). These findings support the association between Hla activity, thrombocytopenia and death among MRSA but not MSSA bloodstream isolates. 

### 2.4. Association of High Hemolytic Activity with High-Risk Source of Infection

We grouped the study isolates according to the source of infection relative to the associated risk of death as reported by Soriano et al [21]. We found a positive trend when correlating hemolytic activity with risk source of infection [median 29.04 HU (IQR 12.08, 59.84) vs. 15.06 HU (IQR 6.25, 34.74), *p* = 0.066] comparing between isolates from high-risk source (n = 33) *versus* intermediate-risk and low-risk sources of bacteremia (n = 67), respectively, but this trend did not reach statistical significance (Figure S1).

## 3. Discussion

The objective of our study was to measure the in vitro level of Hla production and hemolytic activity of *S. aureus* bloodstream isolates and investigate the association of *S. aureus* Hla activity with platelet count and outcome in patients with SAB. We examined a total of 100 *S. aureus* isolates that caused bacteremia chosen to represent equal number of patients who died or survived. From these isolates, in vitro Hla production and hemolytic activity were measured by Western immunoblotting and rabbit erythrocyte-based hemolysis assays using cell-free culture supernatants obtained from bacteria in stationary growth phase. We demonstrated an excellent correlation between hemolysin production in vitro and hemolytic activity. Consistent with previous reports [18,19,20], our findings showed a wide variation in hemolytic activity across bloodstream isolates. 

A strong association between thrombocytopenia, defined as a platelet count less than 150 × 10^9^/L, and 30-day mortality has been previously shown by a retrospective study involving 1052 patients with SAB [22]. In a separate study that included 49 patients with SAB, strains with high Hla expression were associated with thrombocytopenia (platelet count < 100 × 10^9^/L) from the initial blood sample and death in four of nine patients [7]. We extended these findings by analyzing the association of hemolytic activity of the SAB strains and platelet count measured at multiple time points during SAB as well as mortality in a larger patient cohort. 

Notably, we showed a strong association between thrombocytopenia and 30-day mortality and found that platelet count nadir occurred around day 4 from onset of bacteremia. These findings provide clear support of the current clinical practice that considers low platelet count as a poor prognostic indicator in SAB, indicative that platelet count should be followed serially particularly during the early course of bacteremia in line with previous studies that analyzed the association between mortality and multiple time points of platelet count in critically ill patients [23,24].

Previous studies provided mechanistic insights into Hla-mediated platelet dysfunction and depletion in experimental models of *S. aureus* sepsis [6,7,8]. Therefore, we analyzed the relationship between Hla expression of the infecting *S. aureus* isolates and thrombocytopenia and death in patients with bacteremia. Importantly, our results confirmed the relationship between Hla expression and thrombocytopenia and cast a new light on the contribution of methicillin resistance. We found that MRSA bloodstream isolates had higher overall hemolytic activity and that high Hla-producing MRSA strains are significantly associated with thrombocytopenia and death in *S. aureus* bacteremia but the association between high hemolytic activity and poor outcome was not observed with MSSA strains. In line with these results, Coia et al. measured Hla production in a total of 201 isolates of MRSA and MSSA obtained from various body sites and reported significantly higher Hla production in vitro in MRSA relative to MSSA isolates [25]. Our results are in partial agreement with Jacobsson et al. as they found a positive association between Hla hemolytic activity (assessed by measuring the zones of hemolysis in nutrient agar plates using rabbit erythrocytes) and complicated bacteremia, but not mortality [26]. On the other hand, Sharma-Kuinkel et al. reported an inverse association between SAB patients’ outcome and the in vitro Hla production (measured by Western blot and ELISA) and hemolytic activity (measured by rabbit erythrocyte lysis assay) [27]. One potential explanation is the difference in the patient population studied since their population was limited to postsurgical patients and those on hemodialysis whereby our study included a broader range of patient population. Importantly, Sharma-Kuinkel et al. did not analyze their results relative to methicillin resistance which could potentially mask differences between MRSA and MSSA with respect to Hla activity in association with patient outcome as was noted in our study.

Epidemiologic data from a recent retrospective analysis of 92,089 patients indicated that bacteremia caused by MRSA was associated with longer hospitalization, higher rate of readmission with bacteremia recurrence and increased mortality compared to MSSA [28]. Two previous meta-analyses yielded similar findings when comparing mortality rates between patients infected with MRSA and MSSA [29,30]. Our findings suggest differences in virulence of the infecting strains and the Hla-mediated injury to platelets may offer a biologically plausible explanation for the observed difference in mortality between MRSA and MSSA bacteremia. 

It is possible that MRSA and MSSA bloodstream isolates in our cohort differ in genetic backgrounds and that additional virulence factors may be present in MRSA strains that act synergistically with Hla, thereby contributing to the observed difference in patient outcome. Another potential explanation for the difference in hemolytic activity in our MRSA and MSSA strains is the variation between MRSA vs. MSSA in the regulatory component, *agr* system. Agr (the accessory gene regulator) is known to regulate the expression of many virulence genes including Hla [9] and Cheung et al. reported that *agr* enhanced the regulation of methicillin resistance genes (*mecA*, *mecR1*) in community-associated MRSA (CA-MRSA) [31]. Of interest, Otto et al. have previously linked the possibility of increased virulence of CA-MRSA with the increased expression of *hla*, partially because of the enhanced activity of Agr. [32]. Moreover, high Hla protein was detected among highly virulent ST93 CA-MRSA strains while *agr*-deficient strains displayed decreased *hla* expression [33]. Similarly, others have shown increase in the expression of *hla* and *agr* in ST59 CA-MRSA isolates [34]. 

Our study on patients with SAB corroborated published literature on the deleterious effect of *S. aureus* Hla on platelets (e.g., platelet aberrant aggregation and desialylation) in vitro and in experimental models of sepsis [6,7,8] and lend support for future investigations on measuring the virulence phenotype of the infecting strain and therapeutically targeting Hla-mediated effects on platelets to improve patient outcome. Specifically, future studies should examine the feasibility of performing phenotypic assays to measure Hla expression of the infecting *S. aureus* isolates that could be readily adopted into the routine workflow in clinical microbiology laboratory at the time of organism identification. As platelet count measurements are part of routine complete blood count, clinicians should closely monitor platelet count especially during the initial 4 days following onset of *S. aureus* bacteremia relative to Hla expression in the infected strains. Additionally, in vivo studies confirming the modulatory potential of existing therapeutics including antibiotics with antivirulence activity [35] on Hla expression and their benefit in mitigating Hla-mediated platelet dysfunction and depletion could accelerate the translation of our findings to practice.

We acknowledge several limitations in our study. First, we measured the in vitro Hla production of *S. aureus* bloodstream isolates and correlated it with patient outcome; however, it remains unclear how well in vitro Hla production and hemolytic activity correlates with in vivo production during bacteremia. In murine models of pneumonia and skin and soft tissue infection, Berube et al. showed a direct correlation between in vivo Hla production and the degree of tissue injury [36]. Nonetheless, the harmful effects of Hla on platelets are supported by a clear relationship observed between high hemolytic activity of the infecting strains and thrombocytopenia during bacteremia in humans. Furthermore, it is possible that other staphylococcal cytotoxins including the beta, delta and gamma hemolysins and the bi-component leucocidins may have contributed collectively to the observed in vitro hemolytic activity, as their role in the pathogenesis of *S. aureus* diseases has not been fully clarified [37,38]. Finally, we observed very strong signals of high Hla proteins in the Western immunoblot for some of the strains which suggest that the measurements may have reached or exceeded the level of saturation. Therefore, we employed the standard rabbit erythrocyte-based hemolysis assay to capture the variable expression of Hla across our bloodstream isolates. The lack of hemolysis exhibited by our *hla*-deletion strain and the recovery of hemolytic activity of the complemented mutant strain support the validity of the hemolysis assay. 

## 4. Conclusions

In conclusion, our findings show that patients with MRSA bacteremia are more likely to be infected with high Hla-producing strains and are at a higher risk for developing thrombocytopenia and death. Importantly, our study provides support for the future *precision* treatment of infections by phenotyping pathogen virulence and host platelet response to stratify patients who may benefit from treatment that target the Hla-platelet interface thereby improving the outcome of *S. aureus* bacteremia.

## 5. Materials and Methods

### 5.1. Patient and Bacterial Isolate Selection

*S. aureus* isolates and clinical data had been previously collected as part of a large multicenter prospective observational study of adult patients hospitalized for SAB from two affiliated medical centers in Los Angeles, USA. The study was approved by the respective institutional review board at each site; informed consent was waived as the study was observational in design. A total of 100 study isolates were selected to represent equal numbers of patients who died or survived within 30 days of bacteremia onset. Survivors were those who had favorable outcomes: bacterial clearance and clinical improvement by day 4 following onset of SAB and end of therapy success while non-survivors were those whose death was deemed SAB-related in the patient’s medical record and had persistent bacteremia or lack of clinical improvement or worsening on day 4 following onset of SAB. Thrombocytopenia was defined as platelet count < 150 × 10^9^/L at time of SAB onset. Source of infection was grouped based on risk of mortality as previously defined: high (>20%; endovascular, lower respiratory tract, intrabdominal and central nervous system foci), intermediate (10–20%; osteoarticular, soft tissue, and unknown sources), and low (<10%; IV catheter, urinary tract infection, ear–nose–larynx, gynecologic sources, and several manipulation-related sources including digestive endoscopy, arterial catheterization, and sclerosis of esophageal varices) [21]. 

Patients’ medical records were retrospectively reviewed to extract relevant demographics, laboratory and clinical data (see Table 1), and managed using REDCap electronic data capture tools hosted at University of Southern California [39]. Our in vitro analysis included 100 clinical isolates from those patients plus three *S. aureus* control strains: LAC (USA300) *hla* wild type, isogenic Δ*hla* mutant lacking Hla production and Δ*hla*-complemented mutant strains with restored Hla production [40].

### 5.2. In Vitro Measurement of Hla Expression

A single colony of each study isolate freshly grown on a TSA plate was inoculated in 5 mL Tryptic Soy Broth and incubated overnight at 37 °C with shaking at 200 rpm. The OD_600nm_ was adjusted to 0.1 for all isolates and the bacterial suspension was incubated at 37 °C for another 20 h to reach stationary growth phase [41,42]. Then, an aliquot of the culture was removed for CFU determination by plate counting and the remainder was centrifuged at 4 °C and 3100 rpm (Eppendorf Centriguge 5415R) for 10 min to generate cell-free supernatants, which were stored at −80 °C until later analysis. All cell-free supernatants were normalized to 1 × 10^9^ CFU and used in both Western immunoblot and hemolysis assays.

Western immunoblot assay. Ten microliter of cell-free culture supernatants were subjected to SDS-PAGE using 4–20% Tris-Glycine extended gels. Purified Hla (Sigma-Aldrich, St. Louis, MO, USA) was used as controls diluted to in 250 ng, 125 ng and 25 ng/10μL samples. Proteins were then transferred to a PVDF membrane and blocked with 5% BSA in TBST (Tris-buffered saline, 0.1% Tween 20, TBST) for 2 h, then incubated overnight at 4 °C with the primary antibody mouse anti-SA Hla [8B7]-N-terminal (Abcam, Cambridge, UK) diluted 1:2500 in the blocking buffer. After three 5-min TBST washes, the blot was incubated with goat-anti Mouse-HRP (Abcam, Cambridge, UK) as secondary antibody diluted 1:2000 in the blocking buffer for 1 h followed by a 5 min wash in TBST. The signal was developed using TMB (3,3′,5,5′-tetramethylbenzidine) Peroxidase (HRP) Substrate Kit according to the manufacturer’s instructions (Vector kit SK4400, Vector Laboratories, Burlingame, CA, USA). Developed membranes were dried and images were acquired with a Bio-Rad ChemiDoc Touch Gel Imager (Bio-Rad, Hercules, CA, USA) and the quantitative analysis was performed using Image Lab software (6.0.1 version).

Hemolysis assay. The hemolytic activity of Hla was evaluated by measuring the hemolysis of rabbit erythrocytes (Innovative research, Novi, MI, USA). As described previously [27], 100 µL of serially diluted (1:5 up to 1:640 in PBS) culture supernatant was added to a round-bottom polysterene 96-well plate followed by the addition of 100 µL of washed rabbit erythrocytes (1% in PBS). The plate was incubated for 1 h at 37 °C and then centrifuged at room temperature, 220 rpm for 10 min. One hundred µL from each well was transferred to a new flat-bottom polysterene 96-well plate and absorbance was measured at 570 nm. PBS was used as a negative control and 5000 ng purified Hla resuspended in 100 μL PBS was used as a positive control. Hla level (in hemolytic units per ml, HU/mL) was defined as the inverse of the dilution causing 50% hemolysis. All supernatants were tested in duplicates in two independent experiments and the results were averaged.

### 5.3. Data Analysis

The correlation between Hla protein level measured by Western immunoblotting and the hemolytic activity was examined for 61 clinical isolates representing 40 MRSA; 21 MSSA; survivors and non-survivors. The relationship between the hemolytic activity of all 100 SAB isolates relative to methicillin resistance and platelet count at onset of bacteremia, source risk of infection, and 30-day mortality was analyzed.

Statistical analysis was performed using GraphPad Prism (version 9.0). Unpaired t-test, Mann–Whitney tests and Fisher’s Exact tests were used to test continuous and categorical variables where appropriate. Spearman correlation test was performed for correlation analysis. A two-tailed *p*-value of <0.05 denotes statistical significance.

## Figures and Tables

**Table 1:** Comparison of patient characteristics grouped by development of thrombocytopenia and 30-day survival.

Characteristics	Thrombocytopenia (n = 34)	Non-Thrombocytopenia (n = 61)	*p* Value	Non-Survivors (n = 50)	Survivors (n = 50)	*p* Value
Sex (male)	21 (61%)	42 (68.8%)	0.504	31 (62%)	35 (70%)	0.53
Age, y, mean (SD)	58.91 (15.24)	56.84 (18.28)	0.575	62.66 (14.74)	52.60 (17.86)	0.002 *
IV drug use	3 (8.8%)	7 (11.4%)	>0.99	6 (12%)	5 (10%)	>0.99
Alcohol use	5 (14.7%)	12 (19.6%)	0.591	3 (6%)	14 (28%)	0.006 *
Active malignancy	5 (14.7%)	6 (9.8%)	0.498	10 (20%)	3 (6%)	0.071
Liver cirrhosis	8 (23.5%)	5 (8.2%)	0.059	7 (14%)	6 (12%)	>0.99
Renal disease	12 (35.3%)	21 (34.4%)	>0.99	17 (34%)	17 (34%)	>0.99
High risk mortality ^a^	17 (50%)	15 (24.5%)	0.022 *	22 (44%)	11(22%)	0.032 *
Intermediate risk mortality ^b^	10 (29.4%)	20 (32.7%)	0.649	17 (34%)	17 (34%)	>0.99
Low risk mortality ^c^	7 (20.5%)	26 (42.6%)	0.042 *	11(22%)	22 (44%)	0.032 *
MRSA as causative Pathogen	18(53%)	19 (31%)	0.048 *	25 (50%)	15 (30%)	0.065
Platelet count at onset, 10^9^/L (median, IQR)	96.5 (56, 123)	241 (201, 319)	<0.0001 *	128 (87, 229)	221 (170, 315)	0.0002 *
Death	27 (79%)	20 (32.8%)	<0.0001 *			

NOTE: ^a^ endovascular sources, lower respiratory tract, IA, and CNS foci; ^b^ osteoarticular sources, soft tissue sources, and unknown sources; ^c^ IV catheter, UTI, ear-nose-larynx, gynecologic sources, and several manipulation-related sources including digestive endoscopy, arterial catheterization, and sclerosis of esophageal varices. * denotes statistical significance as determined by Student t-test or Fisher’s exact test where appropriate.

## Data Availability

The data presented in this study are available in the Supplementary Materials.

## Supplementary Materials

The following are available online at https://www.mdpi.com/article/10.3390/toxins13100726/s1, Figure S1: Alpha hemolysin hemolytic activity analysis based on the source of bacteremia. Table S1. Characteristics of patients and bacteria isolates.
