# Supplementary material for: Increased Risk of Thrombocytopenia and Death in Patients with Bacteremia Caused by High Alpha Toxin-Producing Methicillin-Resistant Staphylococcus aureus
Source: Toxins (Basel). 2021 Oct 14;13(10):726. doi: 10.3390/toxins13100726 (PMC8537302; doi:10.3390/toxins13100726)
Supplement: Supplementary file 1 [file toxins-13-00726-s001.zip › toxins-1355765 -su-proof from author-send for xml.pdf]

Supplementary Materials: Increased Risk of Thrombocytopenia and Death in Patients with Bacteremia Caused by High Alpha Toxin-Producing Methicillin-Resistant *Staphylococcus aureus*

Fatimah Alhurayri, Edith Porter, Rachid Douglas-Louis, Emi Minejima, Juliane Bubeck Wardenburg and Annie Wong-Beringer

Table S1. Characteristics of patients and bacteria isolates.

Isolate ID	Strain Type	Hla Hemolytic Activity (HU/ml)	Hla Protein level (ng/ 10 µl)	Source of Infection[a]	Intensive Care Unit stay	Day-1 platelets count (10^9/L)	30-day outcome
HH019	MSSA	33.48	_	Low	No	248	Survived
HH020	MRSA	40.2	114.67	Intermediate	Yes	N/A	Death
HH031	MRSA	21.53	102.09	High	Yes	219	Death
HH032	MSSA	18.785	_	High	No	345	Survived
HH035	MRSA	81.52	241.96	Low	Yes	176	Death
HH036	MRSA	10.41	16.59	Low	No	226	Survived
HH037	MRSA	0	0	High	No	281	Survived
HH043	MSSA	41.92	195.75	Intermediate	No	85	Death
HH047	MSSA	10.72	_	Low	No	204	Survived
HH060	MSSA	15.06	111.62	Intermediate	Yes	543	Death
HH070	MSSA	32.74	105.84	High	Yes	315	Death
HH092	MSSA	4.72	0	Intermediate	No	227	Survived
HH111	MSSA	68.77	_	Low	No	432	Survived
HH131	MRSA	11.8	21.32	Low	No	204	Survived
HH140	MSSA	11.77	23.52	Intermediate	Yes	292	Death
HH144	MSSA	5.45	_	Low	No	197	Survived
HH145	MRSA	58.56	235.5	High	No	11	Death
HH150	MRSA	0	0	Low	No	242	Survived
HH151	MSSA	44.44	_	High	No	298	Survived
HH154	MSSA	87.92	_	High	No	254	Survived
HH155	MSSA	0	_	Low	No	211	Survived
HH166	MRSA	24.92	112.1	Low	Yes	111	Death
HH169	MSSA	38.72	_	High	No	168	Survived
HH171	MRSA	21.84	174.64	Low	No	224	Survived
HH178	MRSA	67.81	210.38	High	Yes	117	Death
HH224	MSSA	0	_	Intermediate	No	304	Survived
HH227	MSSA	17.17	_	Intermediate	No	434	Survived
HH243	MSSA	25.66	_	Intermediate	No	204	Survived
HH353	MSSA	0	_	Intermediate	No	252	Death

HH361	MSSA	36.72	213.37	Low	No	100	Death
HH363	MSSA	47.235	_	High	No	398	Survived
HH378	MSSA	65.45	_	Intermediate	No	241	Survived
HH409	MSSA	56.94	_	Low	No	183	Survived
HH419	MSSA	13.35	108.72	Intermediate	Yes	271	Death
LAC082	MRSA	40.33	103.61	Intermediate	No	N/A	Survived
LAC132	MSSA	0	_	High	Yes	N/A	Death
LAC164	MSSA	5.13	4.12	Intermediate	Yes	N/A	Survived
LAC188	MRSA	6.25	23.33	Intermediate	Yes	N/A	Death
LAC475	MRSA	17.65	118.66	Intermediate	Yes	208	Death
LAC502	MRSA	10.5	20.04	Intermediate	No	229	Death
LAC510	MSSA	4.75	11.75	Intermediate	Yes	412	Death
LAC536	MRSA	27.78	162.91	High	Yes	13	Death
LAC545	MSSA	6.78	37.93	Low	Yes	338	Death
LAC550	MSSA	49.32	213.92	High	Yes	106	Death
LAC587	MRSA	34.74	203.82	Low	Yes	96	Death
LAC607	MRSA	24.63	104.1	High	Yes	94	Death
LAC633	MRSA	6.7	49.64	Low	Yes	61	Death
LAC663	MSSA	5.07	_	Intermediate	Yes	18	Death
LAC684	MSSA	13.4	_	Low	Yes	224	Death
LAC698	MSSA	7.65	_	High	Yes	50	Death
LAC699	MRSA	30.96	177.72	Low	Yes	179	Death
LAC727	MRSA	16.33	81.34	High	Yes	270	Death
LAC786	MSSA	0	0	Low	Yes	82	Death
LAC787	MSSA	76.43	242.65	Low	Yes	142	Death
LAC843	MSSA	61.125	_	High	Yes	128	Death
LAC865	MRSA	92.09	214.75	High	Yes	39	Death
LAC919	MRSA	0	0	High	Yes	116	Death
LAC968	MSSA	14.46	10.4	High	Yes	431	Survived
LAC985	MRSA	25.32	187.64	Low	No	154	Survived
LAC986	MRSA	14.865	126.7	High	No	69	Survived
LAC 947	MSSA	138.7	_	Low	No	236	Survived
LAC1010	MRSA	8	10.7	Low	No	218	Survived
LAC1015	MSSA	3.4	_	Low	Yes	323	Survived
LAC1017	MSSA	5.34	_	Low	No	176	Survived
LAC1018	MRSA	56.39	220.59	Low	No	210	Survived
LAC1022	MRSA	3.66	4.47	Low	Yes	39	Survived
LAC1030	MSSA	31.9	129.45	High	No	566	Survived
LAC1056	MSSA	0.59	0	Low	No	313	Survived
LAC1057	MSSA	22.76	147.02	Intermediate	Yes	499	Survived
LAC1060	MRSA	131.25	230.05	High	No	134	Death
LAC1063	MRSA	16.75	66.99	Intermediate	No	198	Survived
LAC1064	MSSA	5.25	17.44	Low	No	169	Survived
LAC1067	MSSA	1.47	_	High	Yes	122	Death
LAC1070	MSSA	18.98	_	Intermediate	Yes	136	Death
LAC1072	MSSA	20.23	_	Intermediate	No	97	Survived

LAC1079	MSSA	13.79	_	Intermediate	Yes	144	Survived
LAC1082	MSSA	29.03	72.43	High	No	130	Survived
LAC1084	MRSA	51.4	208.29	Intermediate	No	111	Survived
LAC1086	MSSA	34.49	_	Intermediate	No	378	Survived
LAC1088	MSSA	29.2	_	Intermediate	Yes	169	Survived
LAC1090	MRSA	53.55	219.42	Intermediate	Yes	93	Death
LAC1094	MSSA	21.59	97.42	Low	No	351	Survived
LAC1105	MRSA	29.53	87.65	High	No	316	Survived
LAC1110	MSSA	9.64	54.01	Intermediate	No	153	Survived
LAC1115	MRSA	3.76	0	Low	No	352	Survived
LAC1117	MSSA	10.25	_	Low	No	175	Survived
LAC1118	MRSA	13.9	74.02	Low	Yes	159	Death
LAC1120	MSSA	13.7	_	Intermediate	No	177	Survived
LAC1122	MSSA	12.99	_	Intermediate	Yes	266	Death
LAC1138	MSSA	18.99	_	Intermediate	No	147	Survived
LAC1139	MSSA	0	_	High	Yes	103	Death
LAC1140	MSSA	7.55	_	High	Yes	49	Death
LAC1152	MSSA	9.69	_	High	Yes	366	Death
LAC1159	MRSA	25.92	154.54	High	Yes	274	Death
LAC1177	MRSA	65.19	216.62	High	Yes	127	Death
LAC1199	MRSA	98.77	220.51	Intermediate	Yes	87	Death
LAC1238	MRSA	75.4	243.39	High	Yes	58	Death
LAC1251	MSSA	82.63	_	Intermediate	Yes	227	Death
LAC1258	MRSA	61.38	222.17	Intermediate	Yes	48	Death
LAC1263	MSSA	103.18	_	High	No	192	Death

NOTE: [a] Mortality risk based on source of infection per Soriano et al.[21]: high risk source of infection include endovascular sources, lower respiratory tract, IA, and CNS foci, intermediate risk source of infection include osteoarticular sources, soft tissue sources, and unknown sources, low source of infection include IV catheter, UTI, ear-nose-larynx, gynecologic sources, and several manipulation-related sources including digestive endoscopy, arterial catheterization, and sclerosis of esophageal varices. Hla hemolytic activity (HU/ml) was assessed by hemolysis assay on rabbit erythrocytes (hemolytic activity shown is the average of 2 independent experiments performed in duplicates). Hla protein level (ng/ 10 µl) was quantified by Western immunoblot assay.

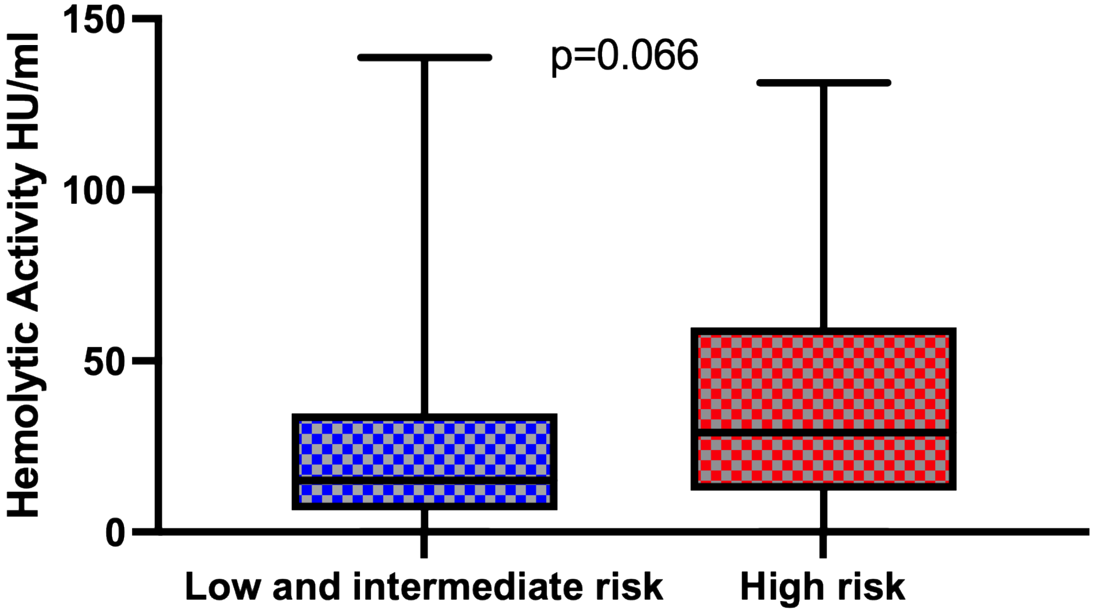

Figure S1. Alpha hemolysin hemolytic activity analysis based on the source of bacteremia

S. aureus isolates obtained from patients were grouped based on the source risk of infection.[21] High risk source of infection include endovascular sources, lower respiratory tract, IA, and CNS foci, intermediate risk source of infection include osteoarticular sources, soft tissue sources, and unknown sources, low source of infection include IV catheter, UTI, ear-nose-larynx, gynecologic sources, and several manipulation-related sources including digestive endoscopy, arterial catheterization, and sclerosis of esophageal varices. Higher hemolytic activity for isolates from high risk vs. intermediate-risk and low-risk sources of bacteremia: [median 29.04 HU (IQR 12.08, 59.84) vs 15.06 HU (IQR 6.25, 34.74), p=0.066]. Box plots represent the hemolytic activity (median value and interquartile range) and whiskers represent min and max values for the respective risk groups. p value as determined by Mann-Whitney test**.**
